# L-phenylalanine attenuates high salt-induced hypertension in Dahl SS rats through activation of GCH1-BH_4_

**DOI:** 10.1371/journal.pone.0250126

**Published:** 2021-04-15

**Authors:** Zhengjun Wang, Chen Cheng, Xiaoyu Yang, Chen Zhang

**Affiliations:** 1 School of Psychology, Shaanxi Normal University and Key Laboratory for Behavior and Cognitive Neuroscience of Shaanxi Province, Xi’an, China; 2 School of Life Science, Shaanxi Normal University, Xi’an, China; 3 College of Animal Sciences, Zhejiang University, Hangzhou, China; 4 School of Life Sciences, Northwestern Polytechnical University, Xi’an, China; Max Delbruck Centrum fur Molekulare Medizin Berlin Buch, GERMANY

## Abstract

Amino acid metabolism plays an important role in controlling blood pressure by regulating the production of NO and ROS. The present study examined amino acid levels in the serum of Dahl SS rats and SS.13^BN^ rats fed a low or high salt diet. We observed that 8 of 27 amino acids responded to a high salt diet in SS rats. Thus, we hypothesized that a defect in amino acids may contribute to the development of salt-induced hypertension. L-phenylalanine was used to treat SS rats with a low or high salt diet. The results demonstrated that L-phenylalanine supplementation significantly enhanced the serum nitrite levels and attenuated the high salt-induced hypertension in SS rats. Low levels of BH_4_ and nitrite and the impaired vascular response to acetylcholine were rescued by L-phenylalanine supplementation. Moreover, increased GTP cyclohydrolase (GCH1) mRNA, levels of BH_4_ and nitrite, and reduced superoxide production were observed in the kidneys of hypertensive SS rats with L-phenylalanine. The antihypertensive effects of L-phenylalanine might be mediated by enhancing BH_4_ biosynthesis and decreasing superoxide production from NO synthase, thereby protecting vascular and kidney function with reduced ROS and elevated NO levels. The present study demonstrated that L-phenylalanine supplementation restored vascular function, suggesting L-phenylalanine represented a potential target to attenuate high salt-sensitive hypertension through GCH1-BH_4_.

## Introduction

High salt-induced hypertension and cardiovascular diseases are important risk factors for death worldwide [1, 2]. Although restricted salt intake is known to be beneficial, excessive salt intake still persists in some areas, especially in North China [3]. Dahl salt-sensitive (SS) rats are widely used for the study of hypertension and show the phenotype similar to human salt-sensitive hypertension [4].

Increasing evidence has indicated that high salt-induced hypertension is a metabolic syndrome [5–7], and this condition could be prevented and influenced by diets in humans and rats [8–10]. Compound protein diets were more protective against hypertension than purified AIN-76A rodent food in SS rats, implying that the amino acid composition of diets contributed to increased blood pressure [10]. However, high dietary protein (30% protein) exacerbated hypertension and renal damage in the kidneys of SS rats [11]. Additionally, a higher intake of branched chain amino acid (BCAA), particularly valine, was associated with a higher risk of incident hypertension [12]. Taken together, these results strongly suggest that the amino acid composition in the diet is closely related to control of blood pressure [13]. It was not clear how dietary amino acids affect blood pressure.

Our previous studies demonstrated that the metabolic features, especially amino acid metabolism, were significantly different between SS rats and SS.13^BN^ rats [14]. In addition, the levels of amino acids were significantly altered in both the plasma and kidney of hypertensive SS rats [15, 16]. The same results were observed in Wistar rats fed high salt diets [17]. Moreover, supplementation with aspartate or L-arginine could increase the levels of NO and attenuate hypertension, implying that improving amino acid metabolism may lower blood pressure induced by high salt diets [18–20]. However, a comprehensive evaluation of amino acids in high salt diets has not been performed.

L-phenylalanine (L-phe), as the essential amino acid, is converted to tyrosine, and effects on blood pressure are potentially due to the changes in tyrosine levels by modulate the production of DOPA (3,4-dihydroxyPhe) and dopamine [21]. Besides, L-Phe can interfere with tetrahydrobiopterin (BH_4_) production, as a cofactor for aromatic amino acid hydroxylation, involved in the relaxation of the endothelium [22]. However, the crucial mechanistic link between L-phe, high salt induced hypertension and its potential impacts on vascular function and oxidation balance is lacking.

We hypothesize that amino acids metabolism may contribute to high salt induce hypertension and the aim of this study was to characterize the metabolic profiles of amino acids in SS rats. We evaluated whether these amino acids, especially L-phenylalanine, which is known to increase GCH activity and BH_4_ biosynthesis, would attenuate the increased blood pressure in high salt-induced Dahl SS rats.

## Materials and methods

### Animals and reagents

The diet used in the present study was obtained from Jiangsu Xietong (Nanjing, China). Dahl salt-sensitive (SS) rats and SS.13^BN^ rats were purchased from Vital River (Beijing) and maintained with 12 h:12 h dark/light and free access to food and water. After adapting for 1 week, 7-week-old male rats were randomized into the low salt diet (0.4% NaCl, LS) and high salt diet (8% NaCl, HS) groups. Systolic blood pressure (SBP) was measured using tail-cuff methods with a computerized system (CODA-4, Kent, USA) as previously described [16]. All animal experiments were performed according to the guidelines of NIH and approved by the Animal Research Committee of Shaanxi Normal University (permit No.2017073).

For assessment of L-phenylalanine supplementation, all 7-week-old male SS rats were maintained on low salt diets and had free access to water. The L-Phe supplementation group was given free access to drinking water with 2.5% w/v L-Phe and low salt diets as described previously [23]. After 4 days of stable baseline blood pressure, the low salt diets were switched to a high salt diet for another 14 days (n = 7–9). At the end of the experiments, the rats were placed in metabolic cages and 24h urine samples were collected. All rats were fasted overnight and sacrificed. For chemical and amino acid analysis, serum and tissue samples were collected and stored at -80°C until analysis. All chemical reagents were obtained from Sigma. A double blind preclinical research was used to reduce the experimental bias.

### Amino acid analysis

For free amino acids, 100 μl of separated serum was extracted with 200 μl of 8% sulfo-salicylic acid solution. After storage at -20°C for 30 min, the supernatants of the samples were collected after 10 min of centrifugation (13000 g at 4°C).

The concentrations of the amino acids were determined using an automated amino acid analyzer (S433-D System, Sykam, German). The analytical detector was set to measure a specific wavelength of 570 nm and 440 nm for proline and hydroxyproline. The buffer solutions, consisting of four different pH values (2.90, 4.20, 8.00), were purchased from Sykam (Germany). The flow rate of the amino acid analyzer was 0.6 ml/min, and the injection volume was 50 μl. The purchased standards of each individual amino acid were used for identification and quantification (external standard method), and the concentrations of individual free amino acid values are expressed in μM. During the running of the samples, a separate delivery pump for the ninhydrin reagent automatically mixes these two solutions, which are kept under nitrogen in the amino acid analyzer. The flow rate for the ninhydrin solution was 0.30 mL/min (130°C).

### Aortic vascular isolation and reactivity measurement

For determination of the effects of L-Phe supplementation on vascular reactivity, aortic rings were isolated with the integrity maintained from all groups at 37°C. Then, vessels were precontracted with phenylephrine. The response of the aortic vessels to ACh was determined by measuring diameter of aortic vessels, as previously described [24].

### Detection of BH_4_, nitrite, superoxide, urinary albumin, and NO synthase activity

Tissues were homogenized in 5 volumes of HEPES buffer (20 μM, pH 7.4) and extracted by centrifugation (10 min, 13000 g, 4°C). The supernatants were collected and measured by BCA protein assays (Thermo). The BH_4_ levels were measured from the aortic vasculature and kidney using a commercial ELISA kit at 480 nm. Quantification of total nitrite was performed as previously reported using nitrate reductase at 550 nm. Superoxide levels were estimated by a lucigenin chemiluminescence-based assay as previously described [25]. Tissue extracts were incubated with 5 μM lucigenin and 10 μM diphenyliodonium (DPI). These data were recorded in 5 consecutive 1-minute measurements and reported in relative light units per minute, normalized by the abundance of proteins. Urinary albumin and NO synthase activity were determined using kits purchased from the Nanjing Jiangcheng Bioengineering Institute (A028-2-1 and A014-2-2).

### Aromatic amino acid and dopamine measurement

L-phenylalanine, tyrosine and dopamine in serum and the kidney were measured as previously described [26]. Briefly, the kidney tissues homogenate (1:10 w/v, PBS, pH = 7.4) or serum were de-proteinated with an equal volume of perchloric acid solution (0.59M) extracted by centrifugation (10 min, 13000 g, 4°C). The supernatant was then used to separate different compounds by HPLC (AntecDECADE II) and quantified using electrochemical detection at 210 nm. HPLC separation was performed using a C18 column (ALF-105 column, 50×1.0mm) and a mobile phase comprising Acetonitrile (2.5%), Octylamine (10 μL/L) and Perchloric acid (0.8 mL/L), pH 2, at a flow rate of 0.75 ml/min. Quantitation of dopamine, L-phenylalanine, and L-tyrosine was performed by comparison with external standards and normalized for total protein.

### Quantitative RT-PCR

Total RNA was extracted from the kidney, and cDNA was synthesized using TaKaRa kits following the manufacturer’s protocol. The expression of GCH1 was analyzed by RT-PCR with SYBR Green PCR Master Mix. Rat GCH1 (forward: 5’-ATTTGTGGGAAGGGTCCA-3’, reverse: 5’-CAGATAACGCTGGCCTCA-3’) was obtained from BGI (Beijing, China). The PCR conditions were as follows: 35 cycles of 94°C for 10 s, 60°C for 20 s, and 82°C for 15 s of fluorescence collection. Expression was quantified by the comparative CT method and normalized to GAPDH with three repetitions.

### Statistical analysis

All data were presented as the mean ± SEM. The normal distribution of amino acids contents were tested with SPSS 25.0 (IBM). A two-way analysis of variance was utilized to determine the differences in parameters between SS and SS.13^BN^ rats on the different diets. Other data were analyzed by two-tailed t-tests. EC_50_ values were compared using Student’s t test. A *p* value <0.05 was considered statistically significant.

## Results

### Effects of high salt diets on amino acids in SS rats

**Fig 1:**
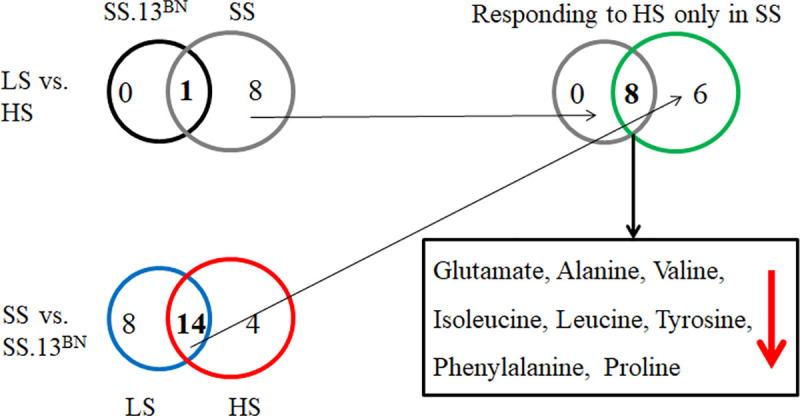
Numbers and overlaps of differentially (*p*<0.05) abundant amino acids in the serum of Dahl SS rats and SS.13^BN^ rats fed a low salt or high salt diet.

Dahl SS rats were significantly hypertensive after they were fed the 8.0% NaCl diet for 2 weeks, with an SBP of 158±7 mmHg compared with 110±5 mmHg on a 0.4% NaCl diet. While the level of blood pressure salt sensitivity was significantly lower in SS.13^BN^ rats (109±4 in low salt and 121±5 mmHg in high salt diet) than SS rats. Compared with low salt diets, high salt diets caused significant reduction of tyrosine in both SS rats and SS.13^BN^ rats (Table 1). Regardless of the diet, 14 of 27 amino acids were significantly different in SS rats and SS.13^BN^ rats (Fig 1). Of the 14 amino acids differentially expressed between SS and SS.13^BN^ rats both before and after high-salt exposure, a set of 8 amino acids responded to high salt only in SS rats: glutamate, alanine, valine, isoleucine, leucine, tyrosine, phenylalanine, and proline, indicating that amino acids were associated with the development of high salt-induced hypertension. Considering the important role of L-phenylalanine in regulating vascular function and ROS levels, we added 2.5% w/v L-Phe for 2 weeks to further elucidate the effects of L-phenylalanine on the development of high salt-induced hypertension in SS rats.

**Table 1:** Data for amino acids in the serum of Dahl SS rats and SS.13^BN^ rats fed a low salt or high salt diet (μM).

	SS.13^BN^-LS	SS.13^BN^-HS	SS-LS	SS-HS
Phosphoserine	70.7±8.6	67.1±10.5	63.0±9.6	61.9±9
Taurine	393.3±33.2	426.7±47.4	564.6±97.9^c^	466.5±59.3
Aspartic acid	17.8±0.7	22.5±4.8	20.7±1.2^c^	19.3±1.1
Threonine	302.6±44.3	300.4±25.2	403.7±46.0^c^	328.4±138.2
Serine	336.5±45.8	389.8±35.5	499.2±57.8^c^	482.6±42.2^d^
Asparagine	69.1±13.1	64.6±6.2	97.3±11.8^c^	84.8±11.3^d^
Glutamate	229±18.3	206.0±37.4	386.1±46.0^c^	283.3±49.6^b^^d^
Glycine	546.3±76.5	627.8±76.0	796.9±101.5^c^	817.5±73.3^d^
Alanine	555.4±225.4	662.3±71.7	1076.9±89.8^c^	849.8±75.2^b^^d^
Citrulline	84.7±18.7	68.2±4.9	86.3±14.8	81.2±3.9^d^
Alpha aminobutyric acid	17.8±1.2	15.8±2.6	27.1±10.2^c^	20.1±1.7^d^
Valine	163.1±28	180.8±22.7	267.8±42.3^c^	214±17.8^b^^d^
Cystine	25.9±0.3	25.1±1.1	38.7±11.7^c^	28.2±1.9^d^
Methionine	74.3±7.7	70.4±6.5	95.8±36.26	87.1±4.9^d^
Isoleucine	112.0±13.9	123.9±10.3	168.9±22.3^c^	142.1±12.2^b^^d^
Leucine	203.5±29.6	236.1±27.01	330.5±38.5^c^	278.4±28.7^b^^d^
Tyrosine	127.1±12.4	111.8±8.4^a^	159.5±15.2^c^	137.9±11.1^b^^d^
Phenylalanine	113.9±12.9	128.6±13.3	178.6±25.1^c^	149.9±11.4^b^^d^
Histidine	42.5±16	64.4±25.8	68.9±11.0^c^	64.6±5.3
3-methylhistidine	1.7±1	5.7±4.0	5.9±2.6^c^	6.3±2.1
1-methyl histidine	23.5±1.9	20.3±2.8	30.5±5.6^c^	29.8±7.1^d^
Tryptophan	107.2±8.2	121.85±16.6	137.2±11.8^c^	119.6±11.1^b^
Ornithine	240.8±27.6	276.74±51.8	361.3±30.5^c^	325.3±24.8
Lysine	340.3±46.1	384.77±42.9	661.2±66.4^c^	588.3±60.8^d^
Arginine	74.2±60.7	38.14±38.9	39.9±45.99	37.7±17.9
Hydroxyproline	220.7±5.9	214.83±4.3	226.1±8.79	224.9±7.3^d^
Proline	185.5±20.6	180.93±9.9	277.6±34.4^c^	231.0±17.6^b^^d^

^a^ *p*<0.05 SS.13^BN^-LS vs SS.13^BN^-HS

^b^ *p*< 0.05 SS-LS vs SS-HS

^c^ *p*<0.05 SS.13^BN^-LS vs SS-LS

^d^ *p*<0.05 SS.13^BN^-HS vs SS-HS. n = 6–8. Data are expressed as mean ± SD.

### Effects of L-Phe on blood pressure

**Fig 2:**
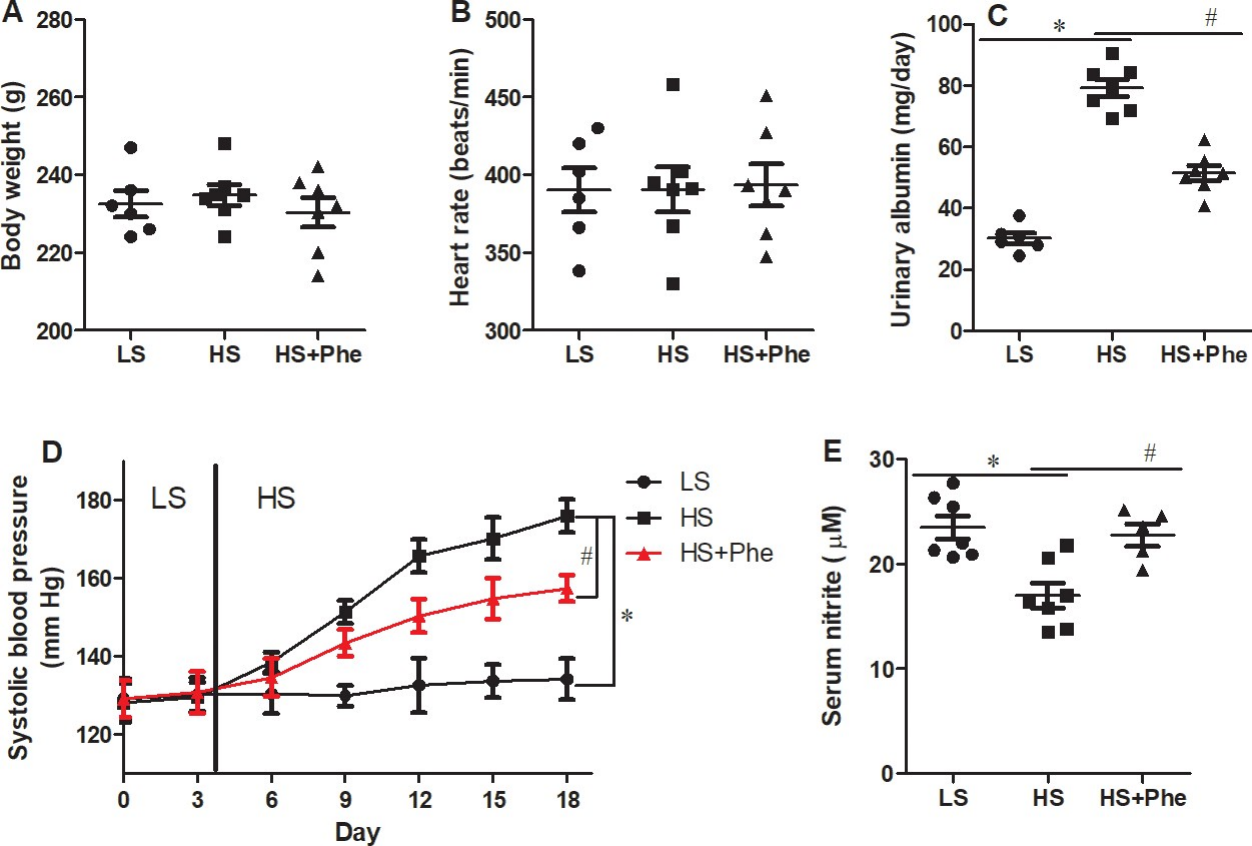
Effects of phenylalanine supplementation on body weight (A), heart rate (B), systolic pressure (C), serum nitrite (D), and urinary albumin (E) in Dahl SS rats fed a low salt or high salt diet. Each value represents the mean ± SEM, n = 6–8, * *p*<0.05 vs. LS group, # *p*<0.05 vs. HS group.

The body weight and heart rate were not different between the groups (Fig 2). Although baseline systolic blood pressure did not differ between the groups, L-Phe supplementation significantly attenuated high salt-induced hypertension in the SS rats (*p*<0.05). By the end of the experiments, systolic blood pressure of the L-Phe supplementation group was 18 mmHg lower than that of the vehicle group. Besides, the urinary albumin was significantly decreased in HS-Phe group than HS group. Significant increase of serum nitrite was also detected in HS-Phe group compared with the hypertensive HS group (Fig 2E).

### Effects of L-Phe on aortic vascular function

**Fig 3:**
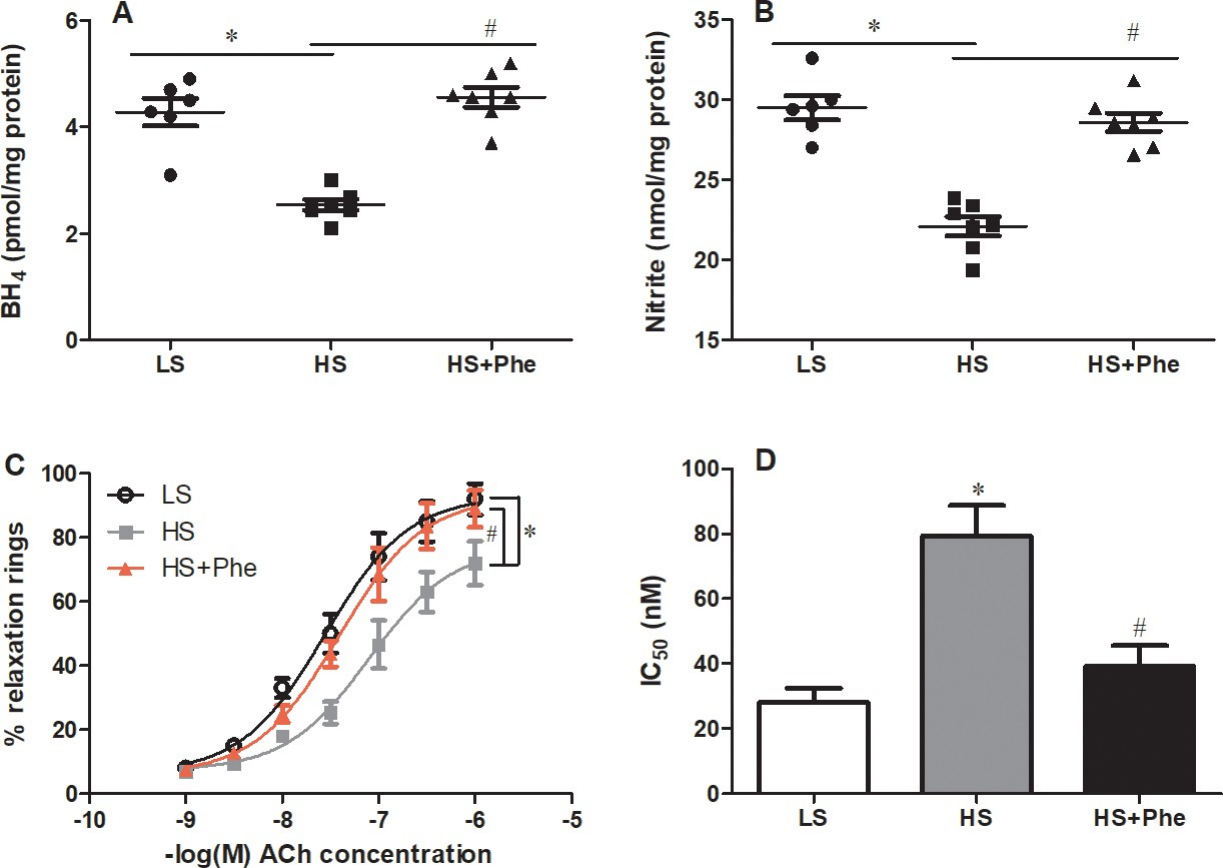
Effects of phenylalanine on BH_4_ (A), nitrite (B), vascular reactivity (C), and the corresponding IC_50_ value (D) in the aortas of Dahl SS rats fed a low salt or high salt diet. Each value represents the mean ± SEM, n = 6–8, * *p*<0.05 vs. LS group, # *p*<0.05 vs. HS group.

Further detailed studies in the vasculature revealed significantly lower BH_4_ levels in SS rats fed a high salt diet than in those fed a low salt diet; these levels were restored to low salt diet values following L-Phe treatment. Consistent with the effects on the BH_4_ levels, L-Phe supplementation significantly increased the nitrite levels in the vasculature of hypertensive SS rats (Fig 3A and 3B). Importantly, endothelial-dependent relaxation induced by ACh in L-Phe treatment group was significantly improved compared with that of the high salt diet control, as reflected by the leftward shift of the dose-response curves and the lower IC_50_ (28.16±7.24 nM in LS group, 79.46±16.38 nM in HS group, and 39.3±10.87 nM in HS-Phe group, Fig 3C and 3D).

### Effects of L-Phe on GTP cyclohydrolase, BH4, nitrite, and superoxide in the kidney

**Fig 4:**
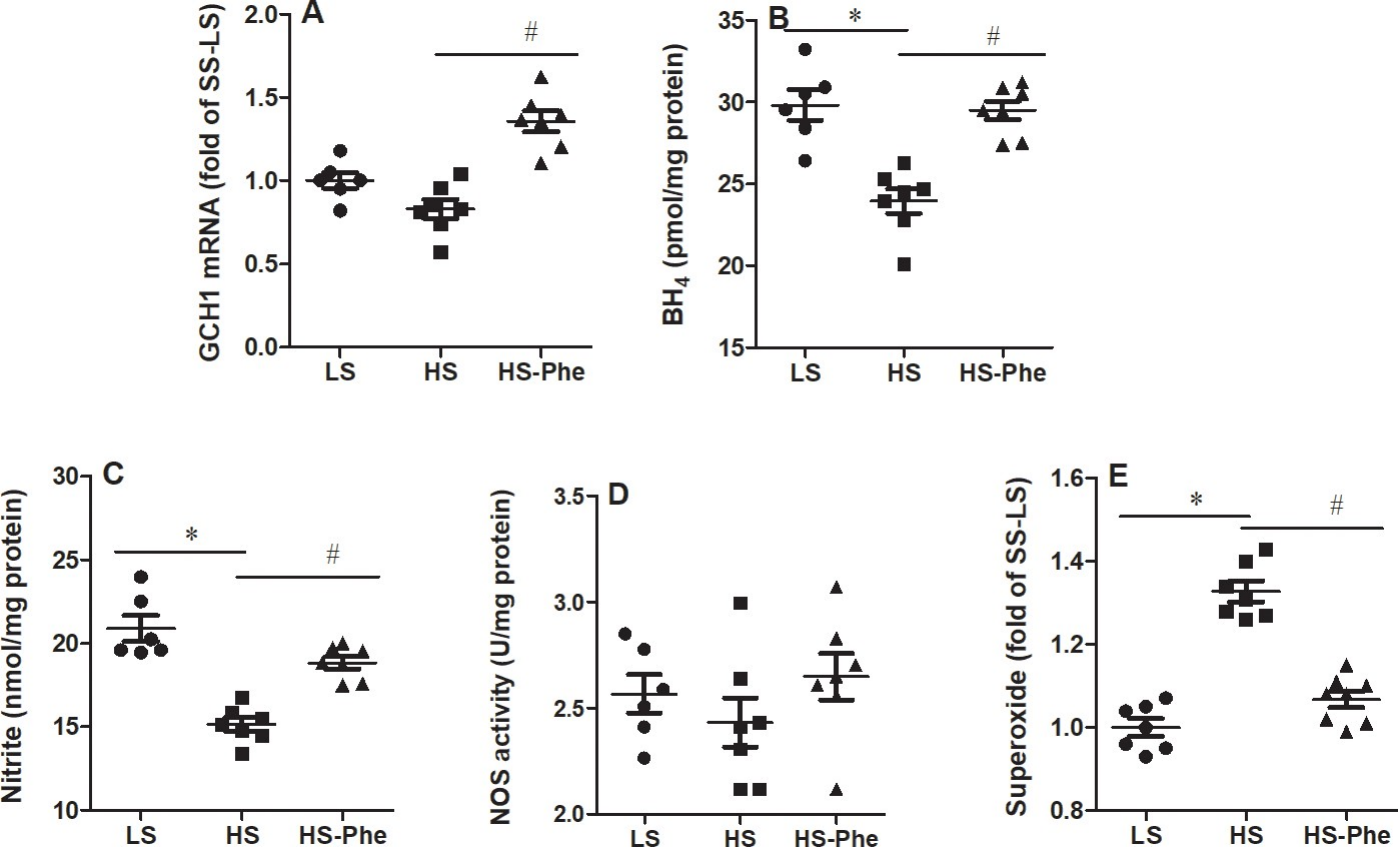
Effects of phenylalanine on GCH1 mRNA (A), BH4 (B), nitrite (C), NOS activity (D), and ROS (E) in the kidneys of Dahl SS rats fed a low salt or high salt diet. Each value represents the mean ± SEM, n = 6–8, * *p*<0.05 vs. LS group, # *p*<0.05 vs. HS group.

It should be noticed that the expression of GTP cyclohydrolase (GCH1) mRNA was no significant changes between LS group and HS group. Whereas, increased levels of GCH1 mRNA was observed in the L-Phe supplementation group compared with the high salt group (Fig 4A). Although levels of nitrite were restored to those on low salt diets, the activity of NO synthase showed no significant changes between the groups (Fig 4B and 4C). Moreover, L-Phe supplementation restored the lower BH_4_ and higher superoxide in the kidney of hypertensive SS rats than those on low salt diet (Fig 4D and 4E).

### Effects of L-Phe on tissue phenylalanine, tyrosine, and dopamine

L-Phe is catalyzed to tyrosine via the enzyme of phenylalanine hydroxylase in vivo. The decreased level of serum L-phenylalanine in the serum of hypertensive rats was reversed in the HS-Phe group (Table 2). Although levels of tyrosine were increased in HS-Phe group, there were no significant differences between HS and HS-Phe in the serum. However, the L-phe/tyrosine ratio was increased flowing with L-Phe supplement, implying that L-Phe was absorbed oral gavage. No significant rise of L-Phe/tyr was observed in both serum and the kidney (Table 2). There was trend of increased dopamine, but it did not reach statistical significance.

**Table 2:** Effects of L-phenylalanine supplementation on levels of L-phenylalanine, tyrosine, L-phe/try, and dopamine in both serum and the kidney of SS rats.

	Serum (μM)			Kidney (pmol/mg protein)		
	LS	HS	HS+L-Phe	LS	HS	HS+L-Phe
L-phenylalanine	175.21±13.64	141.36±11.40*	169.84±13.58^#^	13.02±1.05	12.56±0.79	12.57±1.61
Tyrosine	163.41±10.36	135.72±12.74*	156.53±11.71	7.35±0.75	7.79±0.84	7.76±0.64
L-phe/tyr	1.12±0.16	1.09±0.19	1.12±0.24	1.77±0.62	1.61±0.43	1.73±0.37
Dopamine	0.598±0.047	0.496±0.052	0.547±0.038	10.72±2.81	8.40±1.96	9.84±3.31

* *p*<0.05 vs. LS group

# *p*<0.05 vs. HS group. n = 6. Data are expressed as mean ± SD.

## Discussion

### Altered amino acids in Dahl SS rats

In the present study, we quantitatively analyzed the amino acid contents and showed that only 8 of 27 amino acids responded to high salt in SS rats. Among these amino acids, branched-chain amino acids (Val, Ile, Leu) are structural constituents of proteins that were significantly decreased in hypertensive rats. However, higher BCAA levels were associated with a higher risk of incident hypertension [27]. In the International Study of Macronutrients and Blood Pressure (INTERMAP study), higher intakes of glutamic acid and glycine were related to lower and higher blood pressure, respectively [28]. Although the precise mechanisms responsible for this antihypertensive effect of Pro and Tyr are still unknown, they may involve the inhibition of angiotensin-converting enzyme or production of vasodilators [29, 30]. Compared with those in SS.13^BN^ rats, levels of glycine and methionine were increased in SS rats, which was consistent with previous reports in the plasma and kidney [16, 31]. We proposed that altered amino acids might be responsible, at least in part, for the development of high salt-induced hypertension in Dahl SS rats. Of course, more investigations need to be performed.

### L-Phe altered NO and superoxide levels

Elevated blood pressure has been demonstrated in SS rats with decreased NO [32]. Although there was no significant difference in arginine and NO synthase activity between low salt and high salt diets, the NO levels were significantly decreased in hypertensive SS rats [33], which was due to decreased BH_4_ in the kidney and reduced NO production in the vasculature by uncoupling NOS. Confirming previous findings, we found that L-Phe supplementation reversed the inhibitory GCH1-BH_4_ complex in both the vasculature and kidneys, thus likely contributing to the increased NO production and decreased blood pressure [22, 23]. Furthermore, BH_4_, as an allosteric and essential cofactor for NO synthase, plays a critical role in the stabilization of the NO synthase dimer and increases the affinity of NO synthase for arginine [22, 34]. The decreased L-Phe in the hypertensive SS rats was associated with lower BH_4_, which led to increased sensitivity to high salt diets. Taken together, these data suggested that exogenous phenylalanine may increase BH_4_-dependent NO production, leading to decreased blood pressure in hypertensive SS rats.

The production of superoxide by NO synthase plays an important role in the development of hypertension [35]. BH_4_ has been reported to be a powerful oxidant of O_2_^•^ [36]. The observed decreased superoxide in hypertensive SS rats may result in a shift away from the production of ROS by NOS to the production of NO by the increased BH_4_ with L-Phe supplementation [37]. The effects of L-Phe supplementation on oxidative stress need further study.

### L-Phe improved endothelial function and reduced oxidative damage in the kidney

Endothelial function plays a key role in controlling blood pressure and the development of cardiovascular disease [38]. Our results confirmed that acetylcholine-induced relaxation in high salt diet group was significantly lower than that in SS rats fed low salt diets. However, L-Phe treatment improved vascular function in hypertensive SS rats. These results were consistent with previous studies on treatment with BH_4_ or L-Arg [39, 40]. Thus, we believe that L-Phe attenuates the development of salt-induced hypertension in SS rats by improving endothelial function.

The deficiency in GCH activity was likely contributed to the reduced BH_4_ levels [41]. Although there were no differences in the GCH1 mRNA level between the low and high salt groups, elevated mRNA of GCH1 was observed in HS-Phe group compared with HS group. This finding may be due to insufficient L-Phe contents in hypertensive rats. Correspondingly, this result was particularly relevant to the present study because L-Phe increased the mRNA expression of GCH1. Elevated NO levels and reduced ROS levels may promote sodium excretion and protect the kidney from ischemia [42]. The unchanged BH_4_ level in SS.13^BN^ rats, which was reported previously [41], was consistent with phenylalanine in this strain when fed a high salt diet.

Obviously, phenylalanine hydroxylase also could convert L-phe to tyrosine, which was further catalyzing the conversion of tyrosine to dopamine and adrenaline. Although levels of dopamine were not altered followed with L-Phe treatment, increased blood pressure were attenuated in HS-Phe group indicating that dopamine may be not involved in salt induced hypertension [43]. Consistent with our previous results by GC-MS, no difference of L-phenylalanine and tyrosine were identified in the kidney of SS rats fed on 16wks high salt diets [16]. Previous studies confirmed that L-arginine could restore NO bioavailability through NOS-NO pathway, but L-arginine was not lack of efficacy but also high postinfarction mortality [44, 45], which suggested that L-Phe treatment may be more benefits to attenuate hypertension and to restore vascular function.

We conclude that there were several altered amino acids in hypertensive SS rats that could contribute to salt-induced hypertension. L-Phenylalanine supplementation can directly affect levels of BH_4_ and nitrite, thus attenuating salt-induced hypertension in SS rats through the improved vascular and kidney function.
